# Inflammation and Oxidative Stress in Multiple Sclerosis: Consequences for Therapy Development

**DOI:** 10.1155/2020/7191080

**Published:** 2020-05-12

**Authors:** Valentina Pegoretti, Kathryn A. Swanson, John R. Bethea, Lesley Probert, Ulrich L. M. Eisel, Roman Fischer

**Affiliations:** ^1^Department of Molecular Neurobiology, Groningen Institute for Evolutionary Life Sciences, University of Groningen, 9747 AG Groningen, Netherlands; ^2^Department of Biology, Drexel University, Philadelphia, PA 19104, USA; ^3^Laboratory of Molecular Genetics, Hellenic Pasteur Institute, 11521 Athens, Greece; ^4^Institute of Cell Biology and Immunology, University of Stuttgart, 70569 Stuttgart, Germany

## Abstract

CNS inflammation is a major driver of MS pathology. Differential immune responses, including the adaptive and the innate immune system, are observed at various stages of MS and drive disease development and progression. Next to these immune-mediated mechanisms, other mediators contribute to MS pathology. These include immune-independent cell death of oligodendrocytes and neurons as well as oxidative stress-induced tissue damage. In particular, the complex influence of oxidative stress on inflammation and vice versa makes therapeutic interference complex. All approved MS therapeutics work by modulating the autoimmune response. However, despite substantial developments in the treatment of the relapsing-remitting form of MS, approved therapies for the progressive forms of MS as well as for MS-associated concomitants are limited and much needed. Here, we summarize the contribution of inflammation and oxidative stress to MS pathology and discuss consequences for MS therapy development.

## 1. Introduction

Multiple sclerosis (MS) is a multifactorial autoimmune disease of the central nervous system (CNS) that is characterized by chronic inflammation, demyelination, and axon and neuronal loss. Depending on the location of the demyelinating lesions, MS patients can develop almost any neurological sign or symptom, including motor, sensory, and cognitive impairment [1]. The most common symptoms are numbness, muscle spasms, ataxia, walking difficulties, bladder or visual problems, fatigue, pain, depression, and MS-related dementia [1]. One of the most frequent nonmotor MS-associated symptoms is chronic neuropathic pain (CNP), a long-lasting chronic pain that affects approx. 60% of MS patients and dramatically reduces their quality of life [2, 3]. As a multifactorial disease, the etiology of MS is complex. However, inflammation is a major driver of the pathology. In addition, oxidative stress contributes to tissue injury and promotes existing inflammatory response. Due to the inflammatory nature of MS, targeting of the immune response is the most widely used therapeutic approach. Acute attacks are treated with corticosteroids; however, due to dose-limiting severe side effects, steroids cannot be used for chronic treatment. Currently, 12 immunomodulatory agents are approved as disease-modifying therapies for MS. Adjuvant drugs, such as antidepressants, are typically used to treat MS-associated CNP [4]. However, all of these therapeutics show either a limited efficiency or severe side effects. Further, they do not target all MS symptoms, and treatment options for sensory impairments are limited and often not very effective [2, 4]. Therefore, novel therapeutics that target both motor and sensory MS disease are an urgent medical need. In this review, we will summarize the contribution of inflammation and oxidative stress to MS pathology and discuss current therapeutic developments that may improve MS therapy.

## 2. Multiple Sclerosis

### 2.1. Etiology and Epidemiology

Worldwide over 2.5 million people are living with MS, a number that is constantly growing [5]. Even though MS can develop at any time in life, most people get diagnosed with MS around age 20 to 40 years. Women are more often affected with MS than men, with a two- to threefold higher prevalence and incidence [1]. Similar sex differences were found for MS-related CNP as well as CNP in general [2]. The incidence of MS is impacted by ethnicity, geographical location, and environmental factors, resulting in a variable epidemiology around the world. The general population has a lifetime risk of 0.2% to develop MS. However, siblings of MS patients have a 10- to 20-fold higher risk of developing the disease [6], indicating that genetic factors play an important role for MS development. The first identified mutations that impact MS susceptibility were specific human leukocyte antigen (HLA) variants within the major histocompatibility complex (MHC) gene complex, outlining the important role of the immune system for MS development. However, like other autoimmune diseases, MS is a complex genetic disorder following a polygenic etiology and a multitude of MS-associated genes outside the MHC locus were identified during large genome-wide association studies [7].

### 2.2. Clinical Manifestation

The most common form of MS is the relapsing-remitting course (RRMS), which is dominated by peripheral and central inflammation leading to axonal injury and neuronal loss. Due to the accumulation of neurological signs and symptoms, the RRMS form may evolve years later into the secondary progressive MS (SPMS). Up to 15% of MS patients do not experience relapses and develop directly a primary progressive (PPMS) disease after clinical onset [1]. The mean age of onset is approx. 40 years and is similar in SPMS and PPMS patients [8]. Around 60% of MS patients suffer from CNP which is typically associated with significant disability and depression [2, 3]. MS pain syndromes are divided into primary pain caused by inflammation, demyelination, or neurodegeneration and secondary pain due to indirect consequences of the CNS lesion [9]. MS patients can experience a wide range of CNP symptoms. The most common MS-associated CNP conditions include ongoing dysaesthetic pain in the lower extremities, paroxysmal pain, which is divided into L'hermitte's phenomenon and trigeminal neuralgia, as well as thermal and mechanical sensory abnormalities [2, 4, 9].

### 2.3. Pathology

The central hallmarks of MS pathology are demyelinating plaques within the white and grey matter of the CNS [1]. The location of these lesions within the CNS is quantitatively and qualitatively variable over time and a crucial determinant of the clinical outcome. An inflammatory reaction of autoimmune nature is believed to be the driving force of the demyelinating lesions. Classically, MS is regarded as a T cell-mediated autoimmune disorder [10], and for a long time, it was widely accepted that MS is initiated by an adaptive immune response directed against CNS antigens. Indeed, activated autoreactive T cells infiltrate the CNS, where they upregulate proinflammatory mediators and activate microglia/macrophages, leading to inflammation and demyelination. However, there is now increasing evidence that also B lymphocytes and the innate immune response contribute to the pathogenesis of MS [11, 12]. Other data suggest that oxidative injury and subsequent mitochondrial damage play a pathogenic role for neurodegeneration [13]. Next to the hypothesis that MS is a primary inflammatory disease, in which demyelination and tissue injury are driven by immune-mediated mechanisms throughout all different stages and in all different courses [14], other data indicate that MS is a primary neurodegenerative disease, which is modified and amplified by the inflammatory process [15]. Indeed, oligodendrocyte apoptosis in MS lesions and tissue damage can occur independently of lymphocytes or peripheral macrophages [16], indicating that nonimmune-mediated mechanisms contribute to MS pathology.

Similarly, central inflammation, demyelination, and neurodegeneration lead to the development of MS-associated CNP [2, 17]. Further, data from the rodent experimental autoimmune encephalomyelitis (EAE) model of MS indicate that next to neurodegeneration in the CNS, peripheral nerves undergo major pathologic changes with disease progression [18–20]. Lymphocyte infiltration into peripheral nerves and macrophage activity in the dorsal root ganglion represent a hallmark of peripheral CNP pathology [2], indicating that peripheral inflammation and demyelination may contribute to MS-associated CNP. Indeed, peripheral nerve lesions were observed in MS patients [21]. However, there are no clinical data on association of peripheral neuropathy with occurrence of MS-associated CNP.

## 3. Inflammation in Multiple Sclerosis

### 3.1. Role of Adaptive Immune Cells

The inflammatory lesions within the CNS have been reported to contain CD4^+^ and CD8^+^ T cells, and the meninges in progressive MS contain ectopic germinal centers that include B cells and other immune populations [22], indicating that the adaptive immune system plays a major role in pathogenesis (Tables 1 and 2). The work of Lassmann's group suggests that two types of inflammation occur in MS patients. In acute and relapsing MS, the blood-brain barrier (BBB) becomes leaky and focal bulk invasion of T and B cells into the white matter leads to the classical active demyelinated plaques [10]. The lymphocyte invasion correlates with cytokine activity in the CNS, with disease activity linked to higher expression of inflammatory cytokines. In contrast, the expression of anti-inflammatory cytokines varies more and clinical studies suggest that the phase of RRMS may determine their expression levels. In early stages of MS, a slow but gradually increasing accumulation of T cells and B cells, in the absence of major BBB damage, is observed in the connective tissue spaces of the brain. This second type of inflammation is associated with the formation of subpial demyelinated lesions in the cortex, which are associated with diffuse neurodegeneration in the white or grey matter [10].

Experimental evidence from the EAE rodent model suggests that CD4^+^ T cells are the major drivers of the inflammatory process [22]. Even though a pathogenic role of CD4^+^ T cells in MS would be supported by the genetic association of MS with MHC class II haplotypes and associated molecules [23], the inflammatory cells from the adaptive immune system within MS lesions mainly consist of MHC class I restricted CD8^+^ T cells [24] while MHC class II restricted CD4^+^ T cells are rare and restricted to locations deep within CNS lesions and the cerebrospinal fluid (CSF) [25, 26]. In particular, CD4^+^ T cells are described to contribute to the initiation of the immune response in MS patients, but not to play a major role for the effector stage of CNS inflammation and immune-mediated demyelination and neurodegeneration [22]. Indeed, interferon gamma- (IFN*γ*-) and interleukin-17- (IL-17-) secreting CD4^+^ T cells are believed to be the pathogenic initiators of MS [22], and in MS patients, the increased production of either IFN*γ* or IL-17 is associated with pathology [27]. MS patients also show elevated IL-22 concentration in the CNS, and higher concentrations of this cytokine were observed during the remitting stage [28]. Indeed, secretion of IL-22 promotes CNS infiltration of additional lymphocytes, thus amplifying the inflammatory cascade [29]. The pathogenic role of IFN*γ* in MS is further supported by a clinical MS trial, where IFN*γ* administration exacerbated disease [30]. Similarly, neutralization of IL-17 in MS patients resulted in reduced lesion formation [31]. These clinical data indicate that T helper cells play a role in the induction of CNS autoimmunity in MS. However, it is not completely understood how IFN*γ* and IL-17 initiate or augment disease.

In contrast to CD4^+^ T cells, CD8^+^ T cells are the major lymphocytes found in active MS lesions and CD8^+^ T cells have been identified as potential major contributors to MS pathology. MHC class I expression and presentation are necessary for CD8^+^ T cells to carry out their cytotoxic function. While all cells constitutively express MHC class I, expression was gradually upregulated on astrocytes, oligodendrocytes, neurons, and axons in active MS lesions, making these cells potential targets for CD8^+^ T cells in the context of the disease [32]. Consistent with this, CD8^+^ T cells are able to mediate axonal transection after neuronal MHC class I expression *in vitro* [33]. Further mechanisms of CD8^+^ T cell-mediated neuronal injury may include cytotoxicity by secretion of granzymes, as elevated levels of granzymes were detected in the CSF of relapsing MS patients compared to those in remission [34]. Indeed, histopathological analysis from MS patients revealed that axonal injury correlated with the infiltration of CD8^+^ T cells into lesions [35]. In addition to direct oligodendrocyte death and neuronal injury, CD8^+^ T cells can secrete the cytokines IFN*γ* and IL-17 [36] and may potentiate T helper cell-mediated pathology.

Next to CD4^+^ and CD8^+^ T effector cells, regulatory T cells (Tregs) impact MS pathology. Tregs are master regulators of the immune system that can suppress autoimmunity and contribute to tissue regeneration. In contrast to the effector arm of the immune system, Tregs express TCRs that recognize self-antigens and thereby are activated by self-antigens. In mouse models of MS, Tregs suppress CNS autoimmunity [37, 38], and MBP-reactive, disease-ameliorating Tregs have been identified in mice [39]. In MS patients, Tregs showed functional deficits. Whereas no changes in the frequency of Tregs were observed in the peripheral blood of MS patients, the immunomodulatory function of Tregs is impaired in MS patients [38]. Indeed, whereas Tregs exhibit enhanced migratory characteristics compared to non-Treg cells, this feature is impaired in MS patients [40]. This is in line with data that Treg levels are rather low in the brain tissue of MS patients [41]. In contrast, a highly apoptosis-sensitive Treg subpopulation was observed in the CSF of MS patients [41, 42], indicating that immunomodulatory Tregs might be eliminated by cell death within MS lesions.

Next to T cells, cells from the B cell lineage contribute to adaptive immune inflammation in the CNS of MS patients [25]. Clonally expanded B cells are found in the CSF, the meninges, and the brain parenchyma of MS patients [43]. In early disease stages, CD20^+^ B cells are major components of the lesions, while plasma cells dominate in later stages during lesion maturation and in the progressive disease stage [25]. This is in line with the long-standing observation that immunoglobulin synthesis occurs in the CNS of MS patients [44]. B cells may impact MS through a variety of mechanisms, including the establishment of ectopic lymphoid follicles within the CNS, presentation of antigens to T cells, cytokine/chemokine secretion, and autoantibody production in the CNS [22]. The direct pathogenic role of B cells for MS is supported by data that show that B cells from the CNS of MS patients produce factors that can trigger demyelination and neurodegeneration *in vitro* [45, 46]. In recent years, the essential role of B cells for MS has been validated by successful clinical trials that use anti-CD20 therapy to deplete B cells.

### 3.2. Role of Macrophages/Microglia

Experimental and clinical investigations have demonstrated that microglia- and monocyte-derived macrophages play important roles in MS and EAE [47]. In particular, their interaction and activation by encephalitogenic T cells are critical for inflammatory demyelination in EAE and possibly MS. When fully activated, they can exacerbate neuroinflammation and neuropathology through the production of cytokines, chemokines, and other inflammatory mediators [48]. However, while monocyte-derived macrophages and CNS-resident microglia are heavily implicated in promoting neuroinflammation and degeneration in diseases such as MS, they also hold so-far understudied immunoregulatory, tissue repair, and neuroprotective properties that represent important therapeutic targets for drugs to treat chronic neurodegeneration.

Microglia are CNS resident immune cells. Unlike bone marrow-derived macrophages, they originate from the embryonic yolk sac and represent a self-perpetuating CNS-specific glial cell population [49]. Under physiological conditions, they are important for clearance of apoptotic cells, synaptic pruning, and the formation of mature neuronal circuits during development and are involved in diverse brain processes such as synaptic plasticity, cognition, learning, and memory in the adult [50, 51]. As CNS immune cells, they represent a first line of CNS host defense and are essential for brain protection and homeostasis. They rapidly sense damage- or pathogen-associated signals and become activated to release a host of proinflammatory mediators, such as IL-1*β*, TNF, iNOS, and chemokines, eventually activating and recruiting peripheral immune system cells to infiltrate the CNS. However, microglia immune activity is tightly regulated by inhibitory mechanisms that resolve inflammation to prevent unnecessary tissue damage [52].

In the context of chronic neurodegenerative disease, diverse microglia phenotypes have been detected and their functions are multiplex [53]. On one extreme, they are believed to perpetuate neuroinflammation and disease pathogenesis. Studies in a toxin-induced demyelination model show that microglia are sufficient to drive chronic neuroinflammation in the absence of BBB breakdown and in the absence of significant infiltrating immune cells, a situation similar to that seen in progressive forms of MS [54, 55]. The homeostatic role of microglia in maintenance of neuronal synaptic plasticity is also lost, resulting in synaptic loss in MS and eventually cognitive decline [56, 57]. On the other extreme, there is now compelling evidence that microglia are critical for resistance to EAE onset, a function that involves microglia-specific TNF receptor 2 (TNFR2) [58] as well as tissue repair and recovery, in part through phagocytic clearance of dead cells and debris and in part by production of immunoregulatory mediators [59, 60].

The healthy CNS is also populated by several types of nonmicroglia myeloid cells, including barrier-associated macrophages (BAMs), and CNS dendritic cells (DC) [61]. Both BAMs and CNS DC are mainly located in boundary regions, including perivascular spaces, the meninges, and the choroid plexus [47, 61]. Like microglia, BAMs are long-lived, while CNS DC are bone marrow-derived and short-lived. The precise functions of these nonmicroglia myeloid cells in autoimmune disease are not clear although CNS DC are required for representation of CNS autoantigens to activated T cells, a function critical for the initiation of CNS-directed T cell autoimmune disease [62, 63].

After CNS injury, CNS-resident microglia and macrophages are activated and if additional blood-born monocytes are recruited into the CNS, the BBB is disrupted and neurological symptoms become apparent. In particular, during the effector stage of EAE, monocytes infiltrate the CNS, differentiate into monocyte-derived macrophages, and produce proinflammatory mediators and directly contribute to demyelination. IL-1*β*, an inflammatory cytokine primarily expressed in activated macrophages, monocytes, and microglia, significantly contributes to MS development. IL-1*β* promotes differentiation of T cells into Th17 cells via the STAT3 pathway and thereby promotes and aggravates the inflammatory environment in the CNS [64]. Similarly, monocyte-derived macrophages are mainly found in demyelinated lesions of MS patients [65]. In general, activated monocyte-derived macrophages are thought to be harmful in MS. Indeed, the majority of lesional macrophages belong to the proinflammatory M1 phenotype with only a small percentage of M2 polarized cells [66].

Microglia are the first cells that can take up myelin antigens [67] and become APCs that can activate and intensify adaptive immunity. As antigen-presenting cells (APCs), microglia in turn activate T cells during the course of demyelination and remyelination in MS [56]. Indeed, microglia play a key role for the recruitment of adaptive immune cells to the CNS [68]. After activation, microglia express class I and II MHCs and can activate adaptive immune cells through antigen presentation. In addition, they express costimulatory molecules, such as B7-1 and B7-2, which can interact with CD28 on T cells to stimulate proliferation, differentiation, and cytokine secretion and CTLA4 to promote T cell anergy or apoptosis [69]. Next to their interaction with adaptive immune cells, activated microglia can secrete cytotoxic cytokines and oxidative products, such as ROS and NO radicals in MS lesions thereby promoting oxidative stress and contributing to myelin destruction [56].

Until recently, studies concerning the deleterious disease-inducing effects of chronically activated microglia/macrophages in the CNS have overshadowed the understanding of their powerful endogenous repair potential. Macrophages and microglia show a high plasticity and have been arbitrarily classified into “M1” (proinflammatory) and “M2” (prorepair, anti-inflammatory) phenotypes depending on their activation state, although it is now widely accepted that this classification is hugely oversimplified, particularly for microglia, and only partially reflects the real situation. According to the M1/M2 model, M1 polarized cells are characterized by the release of proinflammatory mediators, such as TNF, IL-1*β*, and IFN*γ*. In addition, they are potent APCs and can activate adaptive immunity. In contrast, M2 polarized cells express a variety of anti-inflammatory mediators, such as IL-4, IL-10, and transforming growth factor-*β* (TGF-*β*), and contribute to immunoregulation [70]. Other data have shown that M2 microglia promote oligodendrocyte differentiation and that microglia depletion impairs remyelination [71]. Multiple intermediate and different microglia/macrophage phenotypes exist that await functional classification.

Indeed, studies aimed at differentiating the effects of microglia and macrophages in the pathogenesis of EAE in mice revealed a significant neuroprotective effect of microglia, via TNFR2, at the onset of disease [58]. Also, administration of a CNS-penetrating inhibitor of soluble TNF in a toxin-induced mouse demyelination model promoted macrophage/microglia phagocytosis of myelin debris and remyelination [60, 72]. Myelin debris is known to be a potent inhibitor of oligodendrocyte precursor cell (OPC) differentiation into myelin-forming oligodendrocytes [73]. In addition, anti-inflammatory mediators secreted from M2 polarized microglia promote remyelination in EAE, for example IL-4, which enhances oligodendrogenesis [74] and suppresses Th1 macrophage reaction, including release of macrophage inflammatory protein (MIP) and activin A, which promotes oligodendrocyte differentiation [71]. Recently, in a mouse model of Alzheimer's disease, a novel disease-associated phagocytic microglial cell phenotype, termed disease-associated microglia (DAM), was associated with restricting neurodegeneration [75].

It is clear that better understanding of the cellular and molecular mechanisms that control the polarization of microglia between proinflammatory to prorepair phenotypes will be critical for the design of drugs that will promote the beneficial functions of these cells and hopefully reverse the inflammatory demyelinating process in MS and provide neuroprotection and CNS repair in other neurodegenerative diseases.

## 4. Oxidative Stress and Mitochondrial Dysfunction in Multiple Sclerosis

### 4.1. Redox Homeostasis and Oxidative Damage

Under physiological conditions, mitochondrial oxidative metabolism produces energy as the end-product of the mitochondrial electron transport chain. Moreover, mitochondria incorporate components of the respiratory transport chain and a set of enzymes, which are the major producers of free radicals within the cell. Free radicals are chemical species with an unpaired electron in their outer orbital which is able to induce reactivity. When oxygen receives an electron, superoxide anion radical (^·^O2-) is formed and the further addition of other molecules generates secondary reactive oxygen species (ROS) such as hydrogen peroxide (H_2_O_2_) and hydroxyl radical (^·^OH). Cellular ROS are also generated in response to endogenous and exogenous stimuli such as cytokines, pathogens, radiations, and xenobiotics [76]. Similarly, to ROS, nitric oxide (^·^NO) is a free radical with an unpaired electron belonging to the reactive nitrogen species (RNS) family.

At moderate concentrations, nitric oxide, superoxide anion, and other ROS play an important role as regulatory mediators in signaling processes. For instance, free radicals and their derivatives are able to regulate vascular tone, sense oxygen tension, enhance the signal transduction from various membrane receptors including the antigen receptor of lymphocytes, and modulate oxidative stress responses in order to maintain redox homeostasis [77]. When cells are challenged by metabolic and temporary environmental stressors, they prevent oxidative damage and maintain redox homeostasis through endogenous feedback mechanisms aimed at continuously balancing electrophiles and nucleophiles [78]. An example of redox signaling is the self-inhibition of neuronal NO synthases which converts to a catalytically inactive ferrous-nitrosyl complex upon NO stimulation [79]. When the feedback loop is disturbed either by a permanent harmful challenge or an inappropriate defense response or inefficient nucleophilic feedback, physiological redox steady state is breached and oxidative damage occurs. In order to avoid this, a complex system of antioxidants is able to effectively support the maintenance of the redox homeostasis. Antioxidants are substances that are able to delay or inhibit oxidation of a substrate at low concentrations. These include both enzymatic and nonenzymatic compounds. Together with cofactors such as copper, zinc, manganese, and iron, enzymatic antioxidants convert dangerous oxidative products to hydrogen peroxide (H_2_O_2_) and then to water. Increased levels of enzymes such as superoxide dismutase (SOD), catalase (CAT), and glutathione peroxidase (GSHPx) increase the number of endogenous antioxidants. The nonenzymatic antioxidants such as vitamins C and E, plant polyphenol, and carotenoids interrupt free radical chain reactions [80]. An important regulator of the antioxidant defense is the nuclear factor erythroid 2-related factor 2 (Nrf2). Nrf2 is a transcription factor that binds to a DNA sequence called antioxidant response element (ARE). When drug-metabolizing enzymes (e.g., cytochrome P450) are activated, Nrf2 detoxifies and eliminates dangerous metabolites by regulating the response against high electrophiles and oxidants [81]. Another important function of Nrf2 is the inhibition of inflammation through inhibition of the NF-*κ*B pathway thereby decreasing cytokine production and oxidative responses [82].

When the antioxidant system is overwhelmed, high levels of free radicals can damage essentially all macromolecules in the cells. ROS may oxidize polyunsaturated fatty acids in lipids by sequestering an electron to increase their stability: an event called lipid peroxidation. A chain reaction is triggered in which a lipid takes an electron from its neighbouring lipid thus leading to the loss of membrane fluidity and elasticity, impaired cellular functioning, and even cell rupture [83]. Moreover, ROS may have dramatic genotoxic actions, which causes the alteration of DNA bases directly contributing to carcinogenesis [84]. It has been estimated that metabolism-generated ROS can induce approximately 10,000 lesions per day in the genome of a human nonneuronal cell [85]. Further, ROS can damage proteins. Even though all amino acids can be targeted by ROS, tryptophan, tyrosine, histidine, and cysteine are particularly sensitive to denaturation [86]. Protein oxidation generates fragmentation at amino acid residues, formation of protein-protein cross-linkages, and oxidation of the protein backbone, which ultimately leads to loss of function. Intracellular pathways are affected by damaged proteins which then contribute to the etiology of different diseases. If protein degradation does not function properly due to altered proteolytic mechanisms, affected proteins accumulate in the cell, developing pathological conditions [87].

### 4.2. Mitochondrial Oxidative Damage and Cell Death

ROS can promote tissue damage by directly activating the apoptosis via the intrinsic mitochondrial pathway, which promotes outer membrane permeabilization and translocation of cytochrome c, apoptosis-inducing factor (AIF), or second mitochondria-derived activator of caspases (Smac/Diablo) from mitochondria to the cytosol. These factors trigger cytosolic apoptotic signaling events or induction of nuclear chromatin condensation and DNA fragmentation by translocation of AIF from the cytosol to the nucleus [88, 89]. To favour this mitochondrial permeabilization and the release of apoptotic signals, the permeability transition pore (PTP) is essential. This is a huge pore spanning the inner and outer mitochondrial membrane, and it is composed mainly of three proteins: the voltage-dependent anion channel (VDAC), adenine nucleotide translocase (ANT), and cyclophilin D (CypD) [90, 91]. The mitochondrial permeability transition pore (mtPTP) is a voltage- and calcium-dependent channel that allows the entry of solutes up to <1.5 kD through the generally impermeable IMM. Alteration in membrane permeability causes depolarization of the transmembrane potential, release of small solutes and proteins, mitochondrial swelling, and loss of oxidative phosphorylation [92]. Evidence shows that there might be both direct and indirect effects of ROS on mtPTP formation. Changes in the membrane conformation can occur due to oxidation of the thiol groups of the IMM that induces disulphide bond and protein aggregation [93]. Moreover, VDAC was shown to regulate the mtPTP and mediate ROS-induced apoptosis. As a matter of fact, VDAC exposes amino acids to the intermembrane space or to the cytosol which are therefore easily accessible for oxidation [94]. Likewise, ANT might also be directly targeted by ROS and this has an effect mainly on its binding to CypD [95]. Nonetheless, recent findings suggested that ROS might affect indirectly the mtPTP. Isolated mitochondria from CypD^−/−^ mice were protected from permeabilization in the presence of H_2_O_2_ or mitochondrial Ca^2+^ overload. In these knockout mice, the mitochondrial membrane potential (MMP) is still generated by TNF, suggesting that CypD involvement is specific to the apoptotic inducer [96].

Although the mechanism and targets of action are still unknown, another important inducer of PTP opening is mitochondrial Ca^2+^ overload. When large quantities of Ca^2+^ accumulate in the mitochondrial matrix, Ca^2+^ interacts with CypD [97]. This interaction could induce the opening of the mtPTP which in turn causes ROS and free fatty acid formation thereby exacerbating the mtPTP opening. Loss of membrane permeabilization causes dissipation of MMP, and if the Ca^2+^ overload persists, the mtPTP will stay open allowing accumulation of solutes in the mitochondrial matrix. Eventually, the outer mitochondrial membrane will rupture releasing the intermembrane space content and proapoptotic signals will leak into the cytoplasm causing death of the cell [98].

It seems that both ROS and Ca^2+^ have key roles in determining oxidative stress-induced mitochondrial dysfunction and cell death. In addition to apoptosis, increased ROS levels lead to other cellular fates including senescence [99], necroptosis [100], and autophagy [101].

### 4.3. Oxidative Damage in Multiple Sclerosis

As previously mentioned in this review, oxidative stress is heavily involved in several MS pathological hallmarks such as myelin destruction, axonal degeneration, and inflammation [102]. In an EAE model, CNS regions characterized by perivascular inflammatory infiltrates show higher mitochondrial dysfunction, fragmentation, and impaired trafficking than other CNS regions [103]. Likewise, active MS lesions show profound mitochondrial protein alterations and DNA deletions in neurons [104]. In these lesions, oligodendrocytes show high levels of oxidized DNA while oxidized phospholipids are preferentially accumulating in axons with disturbed transport. Moreover, the severity of oxidative damage seems to correlate with the extent of inflammation [105]. Furthermore, *in vivo* imaging of EAE-induced axonal damage showed that macrophage-derived ROS can trigger mitochondrial dysfunction and focal axonal degeneration also in axons with intact myelin [106]. This holds true for human multiple sclerosis CNS autopsies where mitochondrial damage is restricted to the lesion area even in the absence of demyelination [106]. In this line, another study shows that accumulation of amyloid precursor protein (APP), a marker for acute axonal damage, occurs not only in active demyelinating but also in remyelinating and inactive demyelinated lesions with a large interindividual variability. APP expression in damaged axons correlates with the numbers of infiltrating leukocytes at the lesion site [107].

Conversely, other studies show extensive oxidative damage to proteins, lipids, and nucleotides in active demyelinated MS regions, specifically in reactive astrocytes and myelin-loaded macrophages [108]. In the same lesions, scavenging activity is also enhanced due to the increased activity of antioxidant enzymes such as SOD1, SOD2, CAT, and heme oxygenase 1 [108] and upregulation of the transcription factor Nrf2 in infiltrating macrophages [109]. In addition, fluorescence life imaging to detect functional NADPH oxidase in an EAE model showed that inflammatory monocytes, activated microglia, and astrocytes are the major sources of oxidative damage within the CNS [110]. Hence, there are discrepancies in literature regarding the cellular localization of oxidative damage within MS and EAE lesions. The reasons for such differences are not clear but they may generate from the high cellular heterogeneity at the lesions' site [105].

Under physiological conditions, neurons, astrocytes, and oligodendrocytes display molecules that bind to microglial receptors, inhibiting their activation state [111]. Decreased expression of these molecules (e.g., myelin CD47) leads to increased microglial activation, which may trigger myelin debris phagocytosis and delivery of neurotrophic factors [111, 112]. Sustained injury, systemic inflammation, proinflammatory cytokine release, and ROS signaling turn microglial physiological functions into toxic inflammatory insults [113]. Taken together, these findings suggest that activated microglia and macrophages are orchestrating tissue injury through their oxidative burst during the development and progression of EAE and MS lesions. Even though a complex antioxidant response is simultaneously triggered, this is insufficient to revert degeneration and apoptotic processes.

The CNS is highly vulnerable to oxidative stress due to several factors such as great energy demand and mitochondrial activity, restricted cell renewal, and large quantity of iron and poly unsaturated fatty acids. Hence, these features increase CNS susceptibility for typical neurodegenerative hallmarks linked with oxidative stress such as impaired mitochondrial function, increased oxidative damage, defect in ubiquitin-proteasome system, changes in iron metabolism, presence of abnormal, aggregated proteins, inflammation, and excitotoxicity [114]. Nevertheless, oxidative damage is not only regulating MS disease within the CNS but it also shapes the immune response developing in the periphery. Firstly, high ROS levels damage the brain endothelium by decreasing its electrical resistance thereby affecting its permeability [115]. In MS patients, nitric oxide metabolites are found upregulated in CSF samples and correlated with relapses suggesting a deleterious role of nitric oxide in inflammatory BBB dysfunction [116]. Furthermore, it has been suggested that interaction of monocytes with the brain endothelium produces ROS facilitating the following intrusion of leukocytes within the CNS [117]. Infiltrating leukocytes are also producing massive amounts of ROS, which induces myelin phagocytosis by activated microglia and macrophages [118], as mentioned above.

The immune system has developed resistance mechanisms and is less sensitive to high ROS levels. Generating H_2_O_2_ and hypochlorous acid enables neutrophils and phagocytes to kill bacteria [119]. ROS signaling is also essential in target cell killing by neutrophils and cytotoxic T cells [120]. Further, T cell receptor activation induces intracellular ROS production [121]. Undoubtedly, ROS signaling is a major contributor in the organism's defense system, but if homeostasis is breached, a vicious circle that comprises inflammation and degeneration will initiate. Similar to MS [122], excessive or sustained ROS levels are involved in the pathogenesis of other neurodegenerative disease [123, 124]. Moreover, the long-standing free radical theory of ageing proposes that ROS are also heavily involved in this natural process and in age-associated diseases [125]. Therefore, therapeutic treatments for MS and other diseases should be aimed at restoring general homeostasis, including redox balance, in order to prevent physiological ROS signaling from being revert.

## 5. Targeting Inflammation and Oxidative Stress to Treat Multiple Sclerosis

### 5.1. Approved MS Therapies

The clinical management of MS addresses three major challenges: (1) prevention of relapses and progressive worsening of disease, (2) handling acute relapses and MS-related symptoms efficiently, and (3) treatment of drug's adverse side effects. Corticosteroids have been used in clinical practice for more than 70 years as immune suppressants. A high-dose intravenous injection of methylprednisolone is the current treatment for acute MS exacerbations. Methylprednisolone immediately decreases CD4^+^ lymphocytes and results in a short-term decrease of IFN*γ* production and chemokine expression levels [126]. This rapid effect has also been linked to transient tightening of the BBB during and shortly after corticosteroid treatment [127]. Even though the resolution of the acute relapse is fast, long-lasting effects of steroid treatment have not been detected.

**Figure 1:**
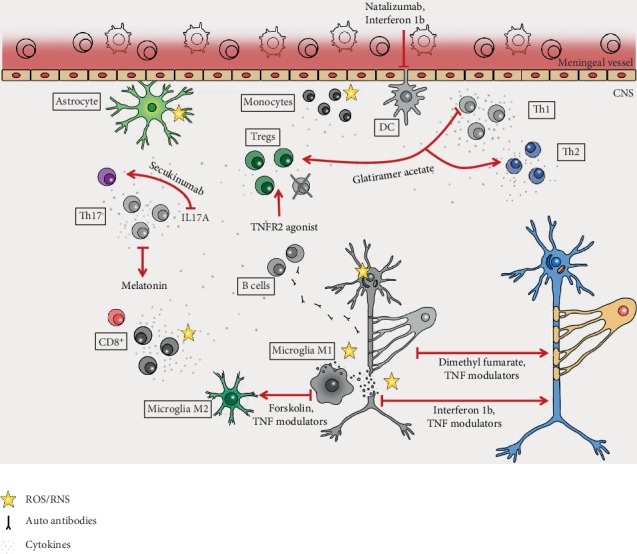
Approved and exploratory immunomodulatory and antioxidant therapeutic strategies to treat MS. MS pathological hallmarks are shown in grey and effect of the therapeutics in color.

However, most of the MS preclinical and clinical studies are mainly focused on the prevention of exacerbations and disease progression. Currently, 12 disease-modifying therapies are approved by the US Food and Drug Administration (FDA) to treat MS (Table 3). Three are injectable medications: interferon beta-1a, interferon beta-1b, and glatiramer acetate; 5 are oral small molecule medications: teriflunomide, fingolimod, dimethyl fumarate, cladribine, and siponimod; 4 are administered via infusion: alemtuzumab, mitoxantrone, ocrelizumab, and natalizumab. In 1993, interferon beta-1b was the first drug to ever be approved for MS, soon to be followed by interferon beta-1a and glatiramer acetate [128]. Since then, interferon beta and glatiramer acetate are typically used as first-line treatment after MS diagnosis (Figure 1). Interferon beta-1a and interferon beta-1b are cytokine derivatives that reduce T cell infiltration into the CNS resulting in alleviated central inflammation [129]. Glatiramer acetate is a random-sized peptide mixture consisting of glutamic acid, lysine, alanine, and tyrosine, 4 amino acids that are enriched in myelin basic protein, a central component of the myelin sheaths [128]. Treatment with glatiramer acetate results in a shift from proinflammatory Th1 cells to anti-inflammatory Th2 cells [130] and an expansion of regulatory T cells [131]. Two disease-modifying drugs are used as second-line treatment in relapsing-remitting MS, natalizumab and fingolimod. Natalizumab is a humanized monoclonal antibody (mAb) against the cell adhesion molecule *α*4-integrin that blocks trafficking of immune cells over the blood-brain barrier into the CNS parenchyma (Figure 1). Fingolimod is a sphingosine-1-phosphate receptor modulator, which sequesters lymphocytes in lymph nodes, preventing them from contributing to an autoimmune reaction, and shifts macrophages into an anti-inflammatory phenotype [129]. Mitoxantrone, teriflunomide, and cladribine are small molecules that inhibit rapidly dividing cells and therefore suppress the replication of T cells and B cells in MS patients [129]. Dimethyl fumarate (DMF) is a small molecule that shifts various immune cell subsets towards an anti-inflammatory state and promotes neuronal survival [132]. Alemtuzumab is a humanized monoclonal antibody (mAb) directed against CD52, a glycoprotein present on the surface of mature lymphocytes, which leads to a rapid, but long-lasting depletion of mature T and B cells [133]. Recently, ocrelizumab, a humanized anti-CD20 mAb, was the first FDA-approved drug for the primary progressive form of MS. Ocrelizumab targets B lymphocytes and kills the cells via antibody-dependent cell-mediated cytotoxicity (ADCC) and, to a lesser extent, complement-dependent cytotoxicity (CDC) [134]. In 2019, the FDA approved siponimod, a sphingosine-1-phosphate receptor modulator and follow-up product of fingolimod, for use in RRMS and SPMS [135]. Indeed, the past 25 years have witnessed substantial developments in the treatment of RRMS. However, approved therapies for the progressive forms of MS, especially PPMS, are limited and much needed.

In general, medications for CNP are limited and often not very effective. Although conventional pain medications can lead to some pain relief, no current therapy provides more than 50% pain relief in the clinic and large randomized and controlled clinical trials for MS-associated chronic neuropathic pain are lacking [4]. Temporary pain relief can be achieved through antidepressants and anticonvulsants. However, these therapies have long-term complications and only a short-term efficacy that leaves patients with untreated and constant pain [2]. As described earlier, TCAs and SSRIs are typically used as first-line drug therapy for MS-associated CNP whereas second-line treatments include opioid analgesics and tramadol [2, 4]. Summarizing, the number of medications to treat MS-associated CNP is limited and their use is often associated with severe adverse events.

### 5.2. Current Developments

#### 5.2.1. Failed Clinical Trials

Despite encouraging results in preclinical disease models, several compounds that modulate the immune system failed in clinical MS trials. A prominent example is targeting of the master proinflammatory cytokine tumor necrosis factor receptor (TNF) with nonselective inhibitors that inhibit both proinflammatory and beneficial functions of this cytokine. Such anti-TNF drugs are blockbuster drugs for use in several autoimmune diseases, such as rheumatoid arthritis, inflammatory bowel disease, and psoriasis [136, 137]. However, the approved anti-TNF therapeutic infliximab and the TNF inhibitor Lenercept failed in clinical trials with MS patients [138, 139], demonstrating that nonselective targeting of TNF is contraindicative in MS.

Despite the clinical success of ocrelizumab, atacicept, a recombinant fusion protein that neutralizes the B-lymphocyte stimulator (BLysB) and A-proliferation-inducing-ligand (April) and inhibits maturation, function, and survival of B cells [140], failed in a randomized, placebo-controlled, double-blind, phase 2 trial. This study had to be terminated early, since atacicept increased relapse rates in MS patients [141], suggesting that the role of B cells and humoral immunity in multiple sclerosis is more complex than currently appreciated.

#### 5.2.2. Antioxidant Therapy

The development of neurodegeneration in MS is a complex process with a multitude of contributing mechanisms, including but not limited to inflammation, primary apoptosis, synaptopathy, mitochondriopathy, and oxidative stress. As described earlier, inflammation and oxidative stress are tightly linked and impact each other. Therefore, next to anti-inflammatory and immunomodulatory treatments, neutralizing free radicals might be a promising therapeutic approach (Table 4). Indeed, DMF was shown to activate antioxidative pathways and to increase expression of the transcription factor Nrf2 [142]. In human oligodendrocytes, DMF stabilizes the cell metabolism resulting in protection from oxidant challenge, providing a mechanism by which DMF may preserve myelin integrity [143] (Figure 1). Even though the mechanism of action of DMF in MS treatment is not well understood, it has been confirmed as a safe antioxidant treatment for MS. In this line, many antioxidant dietary compounds can exert similar functions and boost the beneficial effects of DMFs if used as complementary therapies [144].

In general, antioxidants protect the body against free radicals and are divided into enzymatic and nonenzymatic substances. Enzymes include catalase GPx, GR, and SOD. Nonenzymatic antioxidants may be classified into low molecular weight (e.g., melatonin, vitamins, glutathione, and coenzyme Q) and antioxidant elements (ions) [122]. Melatonin is a neurohormone and important antioxidant that also activates antioxidant enzymes such as SOD, catalase, and GPx [145]. Indeed, melatonin supplementation improved antioxidant defense in MS through upregulation of catalase, manganese superoxide dismutase (MnSOD), and sirtuin 1 (SIRT1), an inhibitor of oxidative stress [146]. Similarly, in a small clinical trial, melatonin supplementation caused a statistically significant increase in SOD and GPx in erythrocytes of SPMS patients. A correlation analysis revealed a positive correlation between SOD levels and the Expanded Disability Status Scale (EDSS) score, both before and after melatonin treatment [145], indicating the importance of antioxidant defense to control MS disability. Another study indicated that levels of melatonin, whose production is modulated by seasonal variations in night length, negatively correlated with MS activity in humans [147]. Further, melatonin treatment ameliorated EAE and directly interfered with the differentiation of human and mouse T cells (Figure 1). In particular, it blocked the differentiation of Th17 cells and promoted expansion of type 1 regulatory T cells (Tr1) [147]. Altogether, these and other studies indicate that melatonin has both immunomodulatory and antioxidant activities. However, the impact of melatonin supplementation on MS disability was modest and larger clinical trials are lacking.

Coenzyme Q10 supplementation for 12 weeks resulted in increased SOD and decreased malondialdehyde A activity in a randomized small clinical trial with RRMS patients, indicating that coenzyme Q10 supplement increases antioxidant enzyme activity and decreases oxidative stress [148]. However, a preclinical study using the EAE model of MS demonstrated that the antioxidant idebenone, a synthetic analog of coenzyme Q10, failed to prevent or attenuate motor disease even when administered preventively [149], suggesting that coenzyme Q10 supplementation may not have an impact on MS disease.

Altogether, this shows that interfering with oxidative stress is a promising therapeutic strategy to treat MS, but might not be sufficient as a single treatment. The combination of antioxidant therapy with other immunosuppressive or immunomodulatory therapies might be superior to current approved therapies (Figure 1).

#### 5.2.3. Selective Modulation of the Immune System

As mentioned before, treatment using nonselective TNF inhibitors failed in clinical trials with MS patients [138, 139]. The failure of these studies might be explained with the pleiotropic actions of TNF. TNF exists in two forms, soluble (sTNF) and transmembrane bound (tmTNF), and activates two receptors, TNF receptor 1 (TNFR1) and TNFR2. Whereas sTNF/TNFR1 signaling promotes inflammation and tissue degeneration, tmTNF/TNFR2 contributes to immune suppression as well as tissue homeostasis and neuroprotection [136, 150]. Blocking all effects of TNF therefore can be counterproductive and exacerbate MS. Given the opposing effects induced by TNFR1 and TNFR2, a more effective therapeutic approach to treat MS therefore is the selective blocking of sTNF/TNFR1 signaling, which leaves TNFR2 signaling functional. Indeed, several studies have shown that neutralization of sTNF/TNFR1 signaling is therapeutic in rodent models of spinal cord injury [151], Parkinson's disease [152], and neuropathic pain [153]. Further, various studies demonstrated the therapeutic potential of sTNF/TNFR1 blocking in the EAE model of MS [154–156]. Therapeutic administration of a selective inhibitor of sTNF in a chronic EAE model rapidly reduced the neurological symptoms of disease, inhibiting spinal cord inflammation and promoting remyelination and neuroprotection [155, 156]. The mechanisms by which sTNF inhibition promote CNS repair were further studied in a cuprizone demyelination/remyelination model where it was found that sTNF inhibits the capacity of microglia to phagocytose and clear myelin debris [60]. Clearance of myelin debris is essential for OPC to be recruited and form new myelin in demyelinated lesions, a function that is critically mediated by tmTNF/TNFR2 [157, 158].

Next to inhibition of sTNF/TNFR1 signaling, specific activation of TNFR2 may hold promise as a new MS therapy. Indeed, TNF promotes proliferation of oligodendrocyte progenitors and remyelination via TNFR2 [157–159]. Further, data from our laboratories indicate that selective agonism of TNFR2 rescues neurons from oxidative stress-induced cell death [160] and excitotoxic cell death [161, 162]. Similarly, TNFR2 activation induces expression of antiapoptotic and detoxifying proteins and protects OPCs against oxidative stress [163]. *In vivo*, TNFR2 agonist administration promoted immunomodulation via expansion [164] and alleviated autoimmune disease [165]. Studies in the EAE model demonstrated that exogenous activation of TNFR2 was therapeutic for motor and sensory disease [166]. Indeed, a recent study in a model of peripheral nerve injury confirmed that TNFR2 is therapeutic for neuropathic pain via an immunomodulatory mechanism [167]. Altogether, these data suggest that selective modulation of TNF-TNFR signaling may hold great promise as a new therapeutic intervention to treat MS [168] (Figure 1).

An important downstream mediator of TNF pathology is the cytokine interleukin 6 (IL-6), which like TNF is found in elevated concentrations in plasma samples [169] and acute and chronic active plaques of MS patients [170]. The pathogenic role of IL-6 was highlighted by data demonstrating that IL-6-deficient animals are fully resistant to EAE [171] and that blocking of IL-6 signaling using an IL-6R-blocking antibody or inhibition of trans-signaling in the periphery led to diminished motor symptoms in EAE [172]. Further, data indicate that IL-6R antagonism is therapeutic for CNP in EAE mice [173], indicating the general suitability of targeting IL-6 to treat MS. Indeed, an exploratory open-label study using the humanized anti-IL-6R monoclonal antibody tocilizumab indicates that RRMS patients receiving tocilizumab had reduced number of relapses, but tocilizumab increased disability in SPMS [174]. Indeed, another study described a patient with rheumatoid arthritis who developed MS during anti-IL-6 therapy [175]. This neuroprotective role of IL-6 is supported by findings that indicate that IL-6 together with TGF*β* restrains Th17 cell-mediated pathology. In particular, stimulation of myelin-reactive T cells with TGF*β* and IL-6 completely abrogated their pathogenic function and Th17 cells failed to upregulate the proinflammatory chemokines crucial for central nervous system inflammation after IL-6/TGF*β* stimulation [176]. This is supported by data indicating that IL-6 contributes to controlling the balance between Th17 cells and Tregs [177]. The clinical importance of IL-17 is further outlined by the first promising clinical results with secukinumab, a fully human monoclonal antibody that neutralizes IL-17A (Figure 1). A randomized proof-of-concept study indicated that secukinumab reduced lesion activity in MS patients and showed a trend toward reduced relapse rates [31]. Further clinical evaluation will reveal whether targeting of IL-17A can be used to treat MS.

Next to direct interference with specific inflammatory cytokines, several preclinical products are developed for MS therapy that promote Treg function. However, laquinimod, an orally available carboxamide derivative that induces Tregs and secretion of anti-inflammatory cytokines as well as direct neuroprotection, failed in a clinical trial. Even though the compound was well tolerated and impacted brain atrophy in a phase III trial, it failed to meet its primary clinical trial goal of slowing progression of RRMS [178]. Clinical evaluation of other therapies that promote Treg function, such as low-dose IL-2 [179], will be necessary to evaluate if correcting Treg function in MS patients is therapeutic.

#### 5.2.4. Microglia Repolarization as a Therapeutic Target

Ablation of microglia impaired development of EAE, indicating the important role of microglia for disease [180]. However, microglia also promote remyelination through the expression of anti-inflammatory molecules, phagocytosis of debris, and repair of tissues [181]. Indeed, microglia were shown to differentiate into different phenotypes during demyelination and remyelination [182]. Whereas M1 microglia contribute to inflammation and oxidative stress-induced oligodendrocyte damage, M2 microglia regulate immune functions and drive oligodendrocyte differentiation during CNS remyelination. In particular, in EAE a switch from a M1- to a M2-dominant response occurred in microglia and peripherally derived macrophages as remyelination started [71]. The important role of M2 microglia/macrophages is supported by experiments demonstrating that *in vitro* OPC differentiation was enhanced in the presence of M2 cell conditioned media. Similarly, blocking M2 activity impaired oligodendrocyte differentiation during remyelination in cerebellar slice cultures and *in vivo* [71]. Indeed, genetic depletion of microglia resulted in inefficient clearance of myelin debris thereby impairing remyelinating processes [183]. Therefore, inhibiting microglia to prevent their proinflammatory and tissue destructive activity might be counterproductive. In contrast, modulation of the inflammatory environment of the lesion, e.g., by repolarization of M1 into M2 microglia, might provide a more promising therapeutic approach. Indeed, the neuroprotective effects of the approved MS drug glatiramer acetate are suggested to be mediated by activated M2 microglia [184]. The sTNF inhibitor XPro1595 [185] also promotes remyelination and neuroprotection in demyelinated lesions by increasing the repair potential of microglia [60]. Several other compounds that modulate microglia/macrophage polarization are currently in preclinical development. The adenylyl cyclase activator Forskolin for example alleviates EAE motor disease by suppressing the expression of CD86 while enhancing M2 macrophage polarization at the site of inflammation [186] (Figure 1). Another example is the clinically approved immunomodulatory agent lenalidomide, which promotes M2 macrophage polarization to regulate CNS autoimmunity resulting in abolished progression of EAE [187].

## 6. Conclusion

MS is a multifactorial disease with a complex etiology. Even though MS is considered an immune-driven disease, several other mechanisms contribute to its pathology, including oxidative stress, immune-independent demyelination, and neuronal cell death. All approved MS therapeutics modulate the immune system thereby suppressing adaptive autoimmunity. However, they are often not effective for all aspects of MS, i.e., sensory deficits, and lead to severe side effects due to unspecific modulation of the immune system. Research of the last decade has shown that selective modulation of the immune system, such as targeting microglia polarization or specific cytokines, might be superior to the currently approved therapies. Two examples are selective targeting of cytokines or microglia. In particular, the cytokines TNF and IL-6, historically considered to be proinflammatory mediators that contribute to MS pathology, contribute to neuroprotection, and neutralization of these cytokines was detrimental in clinical MS trials. Similarly, microglia are cells with a high plasticity and contribute to neurodegeneration, but are also necessary for tissue regeneration. Therefore, selectively targeting the inflammatory activity of these mediators might result in superior therapeutic strategies. Several of the approved MS therapeutics lead to reduction of oxidative stress, and it is hypothesized that this effect contributes to their therapeutic activity. However, different strategies that interfere with oxidative stress failed in clinical evaluation. Nevertheless, these antioxidants may prove to be beneficial as cotreatments with anti-inflammatory reagents resulting in superior clinical outcome. Altogether, several promising novel therapeutic strategies that specifically target components of the neuroinflammatory process are currently under preclinical and clinical evaluation and may lead to the development of novel MS therapeutics with better activity and safety profiles.

## Figures and Tables

**Table 1:** Overview of the cellular immune contribution to MS pathology.

Cell	Effect	Role
B cells	Proinflammatory	Antibody production, antigen presentation to T cells, cytokine production Participate in the adaptive immune response [25, 43, 45, 46]
CD4^+^ T cells	Proinflammatory	Recognize and proliferate in response to autoantigens, cytokine production, drive the inflammatory process [22, 26]
CD8^+^ T cells	Proinflammatory	Recognize and proliferate in response to foreign/self-antigens, target cell cytotoxicity, main T cell type present in MS lesions [24, 26, 32]
CNS dendritic cells	Proinflammatory	Involved in (re)presentation of MS autoantigens to active T cells [61–63]
Macrophage (M1)	Proinflammatory	Activated in response to T cell infiltration, phagocytosis, antigen presentation to T cells, production of proinflammatory cytokines, chemokines, and nitric oxide, increase neuropathy, represent the majority of macrophages in active MS [48, 66]
Macrophage (M2)	Anti-inflammatory	Phagocytosis, antigen presentation to T cells, production of anti-inflammatory cytokines, involved in repair mechanisms, low numbers found deep inside MS lesions [66]
Microglia	Both	CNS surveillance and host defense, activated in MS lesions, production of cytokines, roles in tissue damage and repair, but differential roles to infiltrating macrophages not well understood [48, 52, 56, 58, 67, 71]
T helper (Th17) cells	Proinflammatory	Significant initiator of inflammation in CNS [64]
Tregs	Anti-inflammatory	Suppress autoimmunity, low expression in MS brain tissue [37, 38, 41, 42]

**Table 2:** Overview of cytokines and other immune proteins that contribute to MS pathology.

Protein	Type	Effect	Role
Activin A	Cytokine	Anti-inflammatory	APC costimulation of T cell responses [71]
B7-1	APC membrane protein	Proinflammatory	APC costimulation of T cell responses [69]
B7-2	APC membrane protein	Proinflammatory	APC costimulation (inhibitory) of T cell responses [69]
CTLA4	Receptor	Anti-inflammatory	T cell produced cytokine, associated with increased pathology [22, 27, 36]
IFN*γ*	Cytokine	Proinflammatory/inflammation associated	Anti-inflammatory cytokine, produced by macrophages, Th2 cells, and regulatory T cells, promotes expression of immune-modulating Tregs [70]
IL-10	Cytokine	Anti-inflammatory	Produced by T cells, neutrophils, and other immune cells, associated with pathogenesis [22, 27, 36]
IL-17	Cytokine	Proinflammatory	Proinflammatory cytokine produced by activated macrophages/microglia [52, 64]
IL-1*β*	Cytokine	Proinflammatory	Produced during inflammation, proinflammatory and tissue protective functions, barrier maintenance [29]
IL-22	Cytokine	Proinflammatory	Produced by immune cells including T cells and type M2 microglia and macrophages Pivotal role in shaping immune responses [70]
IL-4	Cytokine	Anti-inflammatory	Produced by proinflammatory macrophages, high levels in CSF associated with greater severity of MS [52]
iNOS	ROS-related enzyme	Proinflammatory	Multifunctional cytokine involved in immune regulation, inflammation, and repair Produced by T cells and type M2 microglia and macrophages, some role in modulation of Th17 cell differentiation [70]
TGF-*β*	Cytokine	Anti-inflammatory	Multifunctional cytokine with proinflammatory and cytotoxic (soluble TNF) and beneficial (tmTNF) effects in the CNS [52]
TNF	Cytokine	Proinflammatory	APC costimulation of T cell responses [69]

**Table 3:** List of FDA-approved disease-modifying therapies to treat multiple sclerosis, adapted from [128, 129, 188, 189].

Drug	Route of administration	Drug class	Mechanism of action	Treatment strategy	Main possible side effects when compared to placebo	Approved indication
Interferon beta-1a	Injection	Protein biologic	Immunomodulatory	First line	Injection site reaction, influenza-like symptoms, lymphopenia, depression	RRMS
Interferon beta-1b	Injection	Protein biologic	Immunomodulatory	First line	Injection site reaction, influenza-like symptoms, lymphopenia, depression	RRMS
Glatiramer acetate	Injection	Peptide polymer	Immunomodulatory	First line	Injection site reactions, vasodilatation, rash, dyspnea, chest pain	RRMS
Teriflunomide	Oral	Small molecule	Immune suppressive	First line	Hepatotoxicity, alopecia, diarrhea, influenza, nausea, and paresthesia	RRMS
Fingolimod	Oral	Small molecule	Immunomodulatory	First line	Headache, liver transaminase elevation, diarrhea, cough, influenza, sinusitis, pain	RRMS
Dimethyl fumarate	Oral	Small molecule	Immunomodulatory	First line	Flushing, abdominal pain, diarrhea, nausea	RRMS
Cladribine	Oral	Small molecule	Immune suppressive	First or second line	Upper respiratory tract infection, headache, lymphopenia	RRMS, SPMS
Siponimod	Oral	Small molecule	Immunomodulatory	First line	Headache, hypertension, transaminase increases	RRMS, SPMS
Alemtuzumab	Infusion	Humanized mAb	Immune suppressive	Second or third line	Infusion reactions, infections, rash, headache, pyrexia	RRMS
Mitoxantrone	Infusion	Small molecule	Immune suppressive	Second or third line	Nausea, alopecia, urinary tract infection, cardiotoxicity, menstrual disorders	RRMS, SPMS
Ocrelizumab	Infusion	Humanized mAb	Immune suppressive	First or second line	Infusion reactions, skin and respiratory tract infections	RRMM, PPMS
Natalizumab	Infusion	Humanized mAb	Inhibits immune cell trafficking into CNS	Second line	Delayed infusion reactions, progressive multifocal leukoencephalopathy (PML), hypersensitivity, immunosuppression/infections, headache, fatigue	RRMS

**Table 4:** Antioxidant complementary therapies and their relevance for MS. Complementary antioxidant therapies for MS were reviewed in detail in [144].

Compound	Specification	Antioxidant characteristics	Relevance for MS
Coenzyme Q10	Coenzyme	Energy transfer molecule, cofactor in mitochondrial electron transport chain	Increases SOD and decreases malondialdehyde A in RRMS patients; synthetic analog has no effect on EAE
Curcumin	Natural pigment	ROS, RNS, and peroxyl radical scavenger; it also modulated GSH, catalase, and SOD activities [190]	Decreases EAE clinical severity, demyelination, and inflammation in the spinal cord and IL-12 production by macrophages/microglia through Janus kinase-STAT pathway [191]
Melatonin	Neurohormone	Activates SOD, catalase, and GPx	It increases SOD and GPx levels in erythrocytes of SPMS patients. Its levels negatively correlate with lesion activity. It ameliorates EAE symptoms, blocks Th17 differentiation and promotes Tr1 expansion.
Vitamin A	Essential nutrient (retinoic acid)	Hydrophobic polyene chain quenches singlet oxygen and neutralizes thiyl radicals stabilizing peroxyl radicals [192].	Serum levels are low in MS patients during relapses [193]. It increases TGFbeta and FoxP3 expression in PBMCs in Avonex-treated RRMS patients [194]. Retinoic acid inhibits cytokine production by Th17 cells in EAE [195].
Vitamin C	Essential nutrient (ascorbic acid)	Scavenges ROS and RNS [196]	Serum levels are low in MS patients during relapses [193]. It promotes OLGs generation and remyelination [197].
Vitamin D	Essential nutrient	Inhibits iron-dependent lipid peroxidation [198]	Serum levels are low in MS patients with elevated relapse frequency [199]. It diminishes risk of MS although the therapeutic value is still debated [200].
Vitamin E	Essential nutrient (alpha-tocopherol)	Peroxyl radical scavenger [201]	Serum levels are low in MS patients during relapses [193]. During IFN*β* treatment, decreased MRI activity in RRMS patients is associated with higher levels of alpha-tocopherol [202]. It decreases IFN*γ* production, inflammation, and demyelination in the spinal cord of EAE mice [203].
